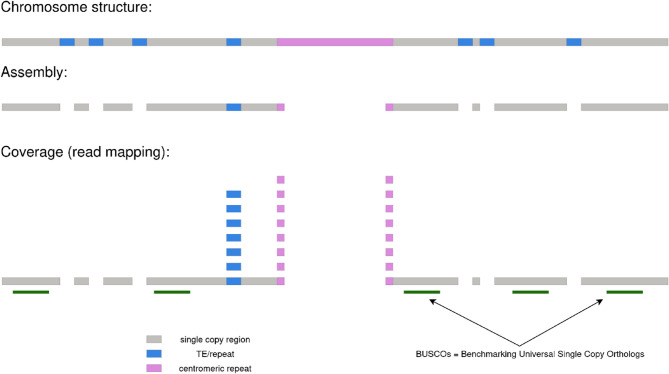# Correction: Mapping-based genome size Estimation

**DOI:** 10.1186/s12864-025-11836-y

**Published:** 2025-07-14

**Authors:** Shakunthala Natarajan, Jessica Gehrke, Boas Pucker

**Affiliations:** 1 Plant Biotechnology and Bioinformatics, Institute of Plant Biology & BRICS, TU Braunschweig, Mendelssohnstrasse 4, 38106 Braunschweig, Germany; 2 Molecular Plant Sciences, Institute for Cellular and Molecular Botany, University of Bonn, Kirschallee 1, 53115 Bonn, Germany

**Correction:** ***BMC Genomics*** **26, 482 (2025)**

**https://doi.org/10.1186/s12864-025-11640-8**

Following publication of the original article it was reported that there was an error in Fig. 1. The incorrect and correct versions of Fig. 1 are given below, and the original article has been updated.

**Incorrect Fig. 1**.


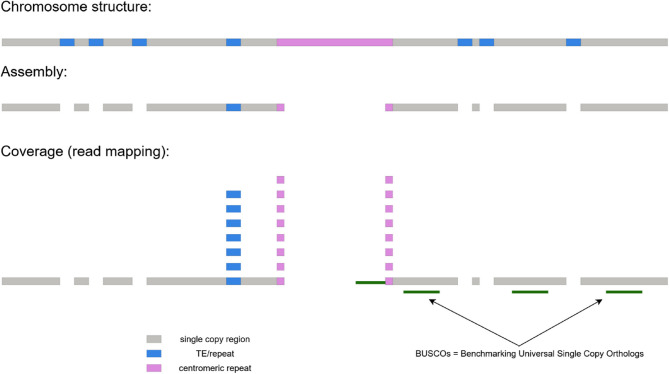


**Correct Fig. 1**.