# Supplementary figures and images for: Mapping-based genome size estimation
Source: BMC Genomics. 2025 May 14;26:482. doi: 10.1186/s12864-025-11640-8 (PMC12079912; doi:10.1186/s12864-025-11640-8)

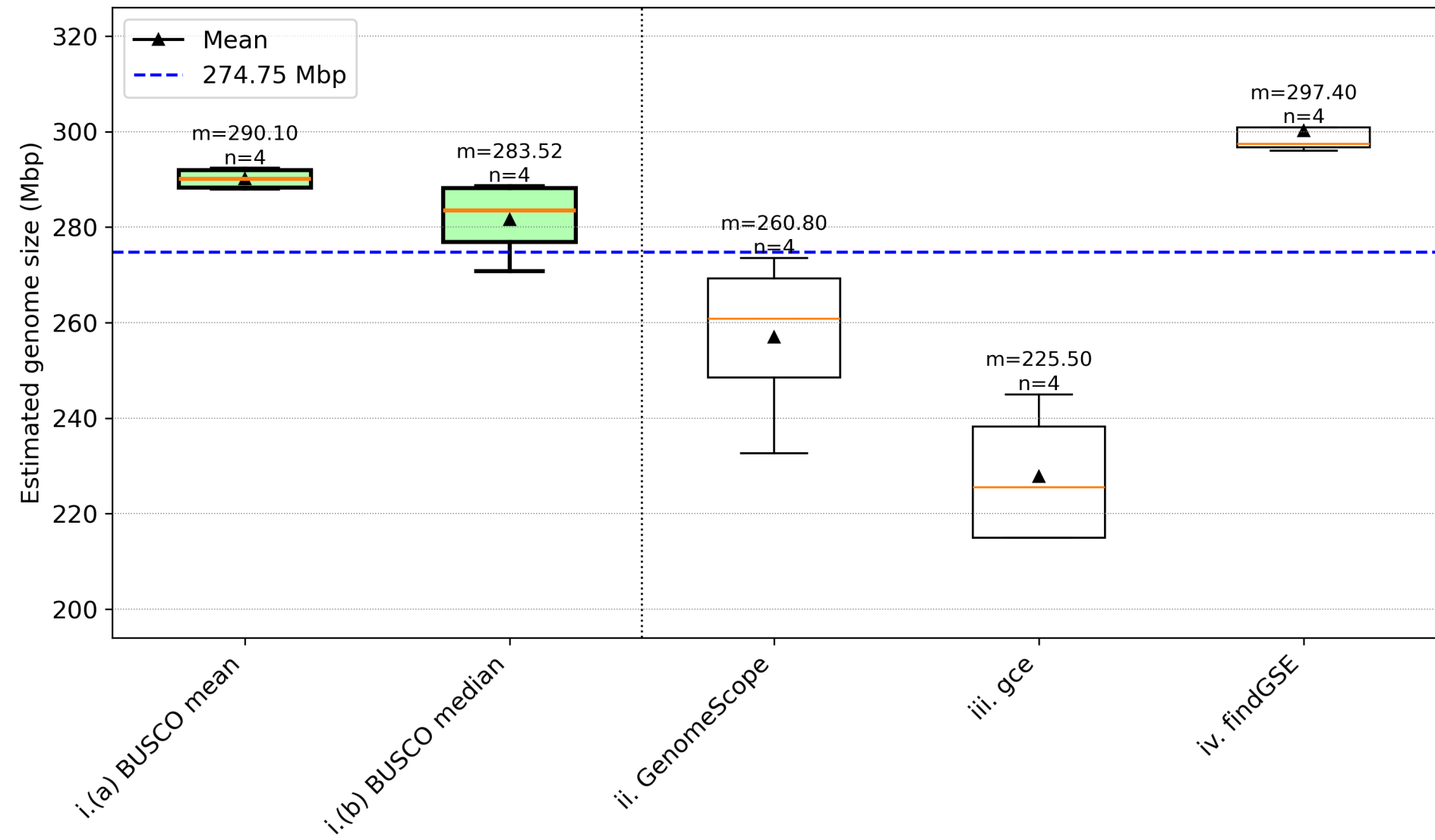

Supplement: Supplementary file 9 — Supplementary Material 9 [file 12864_2025_11640_MOESM9_ESM.pdf]

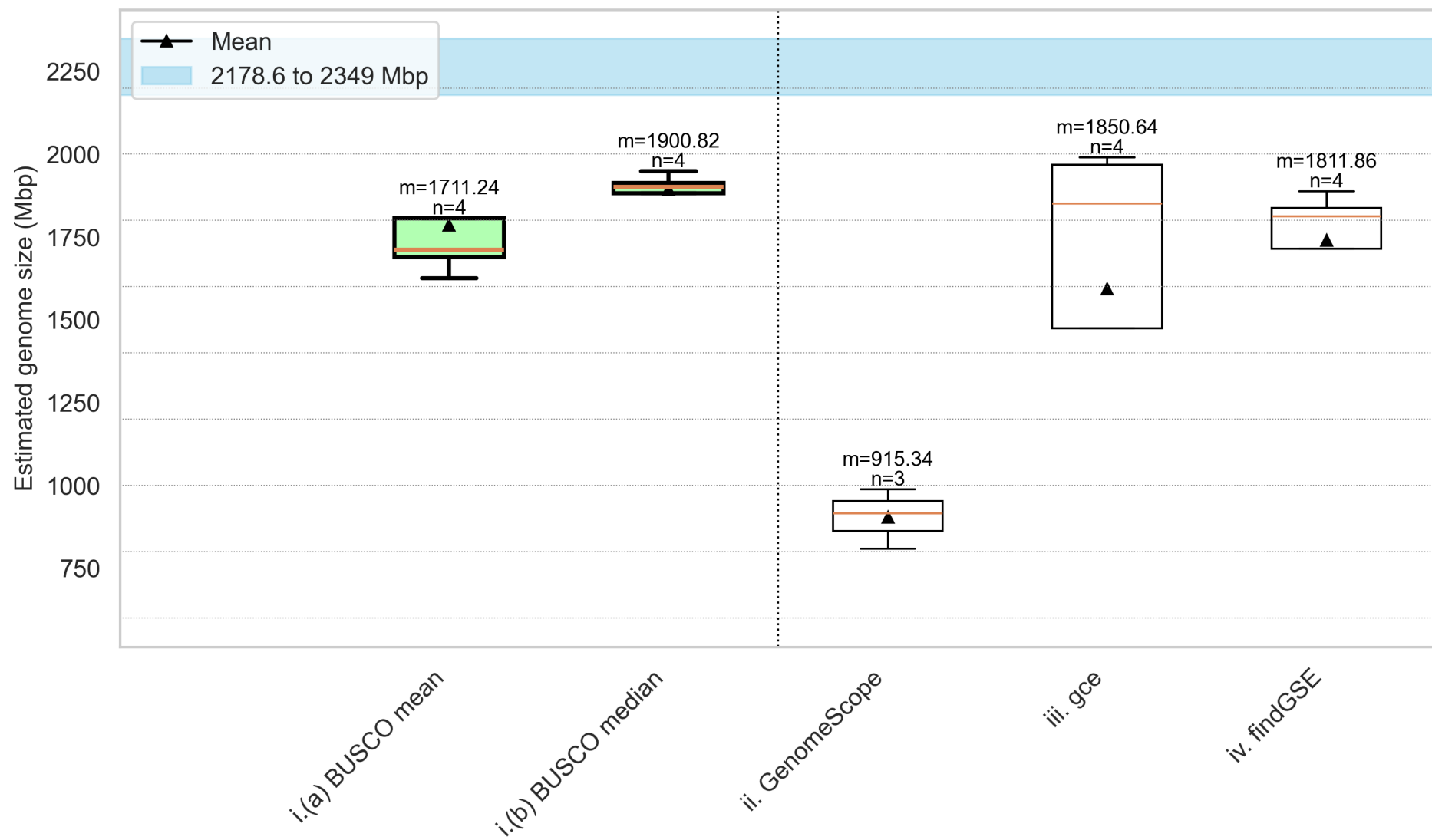

Supplement: Supplementary file 10 — Supplementary Material 10 [file 12864_2025_11640_MOESM10_ESM.pdf]

Estimated genome size (Mbp)

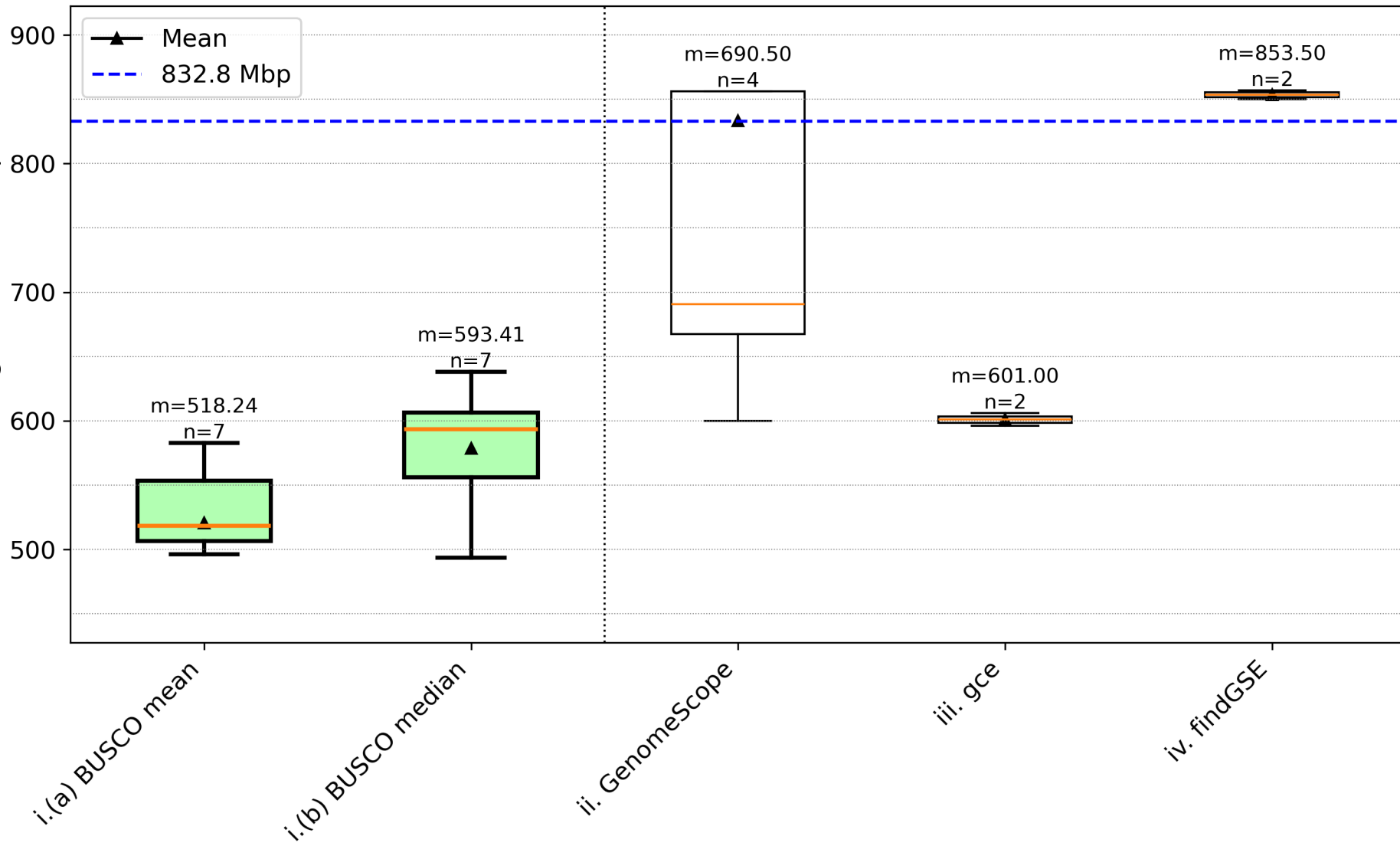

Supplement: Supplementary file 11 — Supplementary Material 11 [file 12864_2025_11640_MOESM11_ESM.pdf]

Estimated genome size (Mbp)

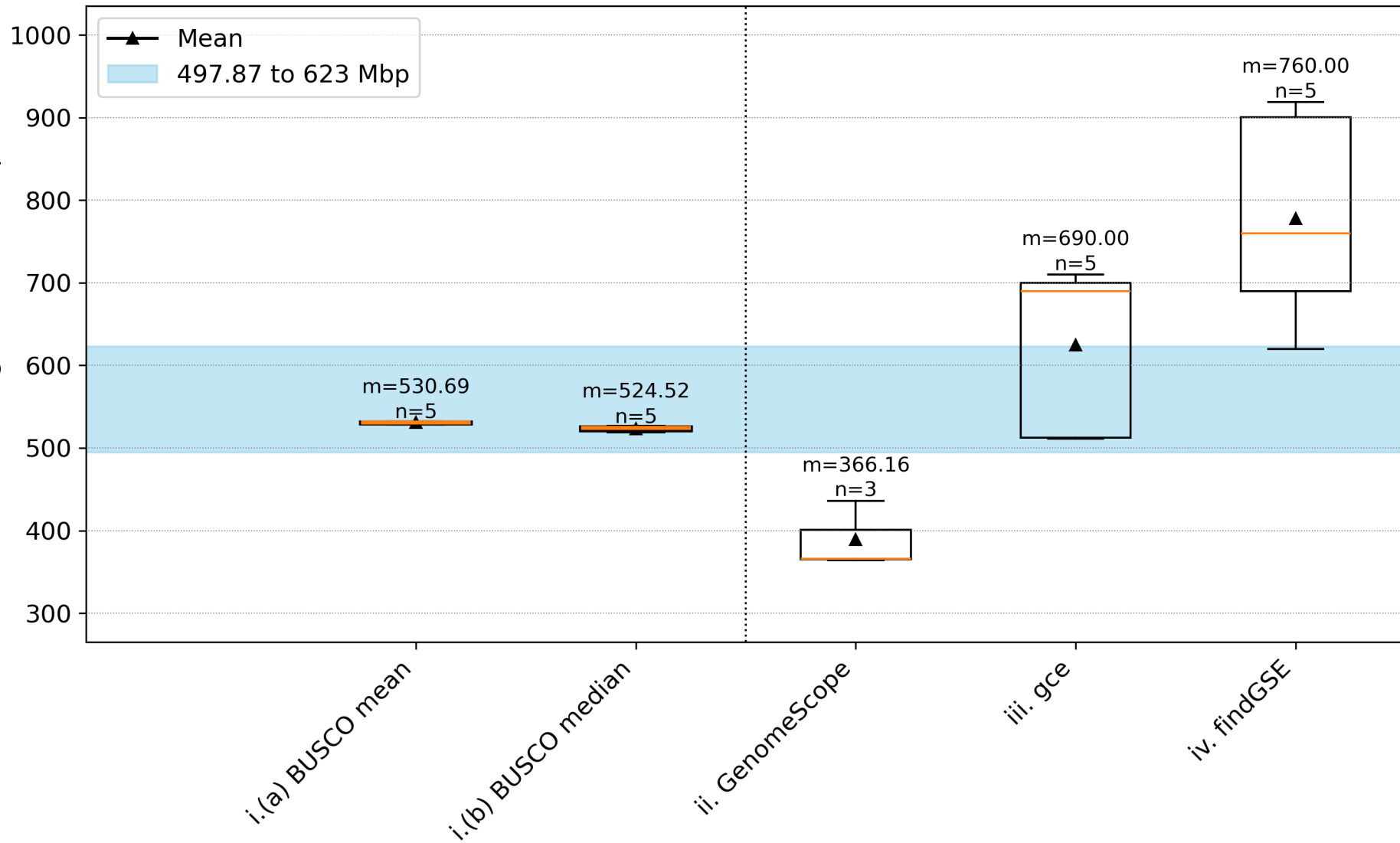

Supplement: Supplementary file 12 — Supplementary Material 12 [file 12864_2025_11640_MOESM12_ESM.pdf]

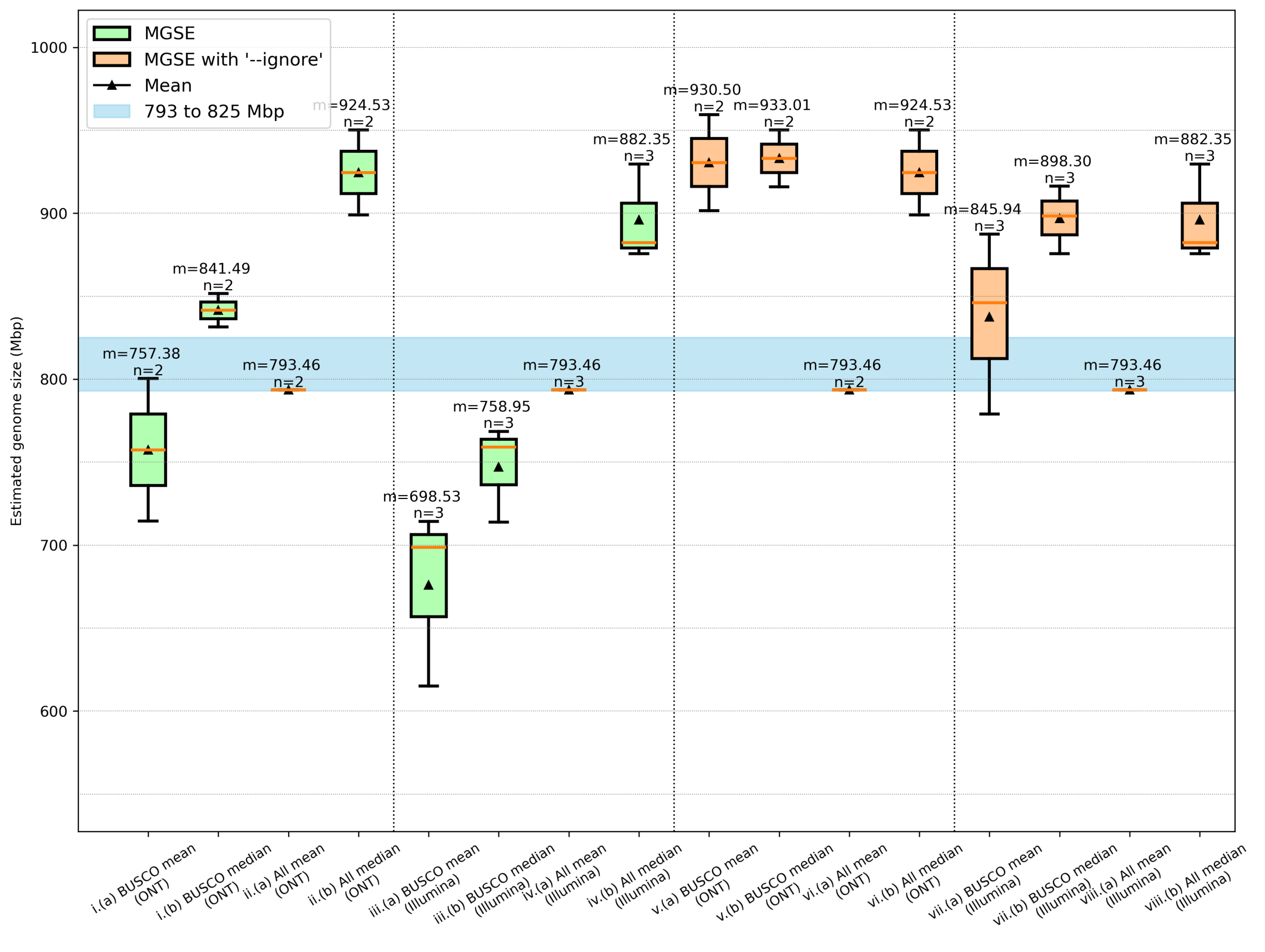

Supplement: Supplementary file 13 — Supplementary Material 13 [file 12864_2025_11640_MOESM13_ESM.pdf]

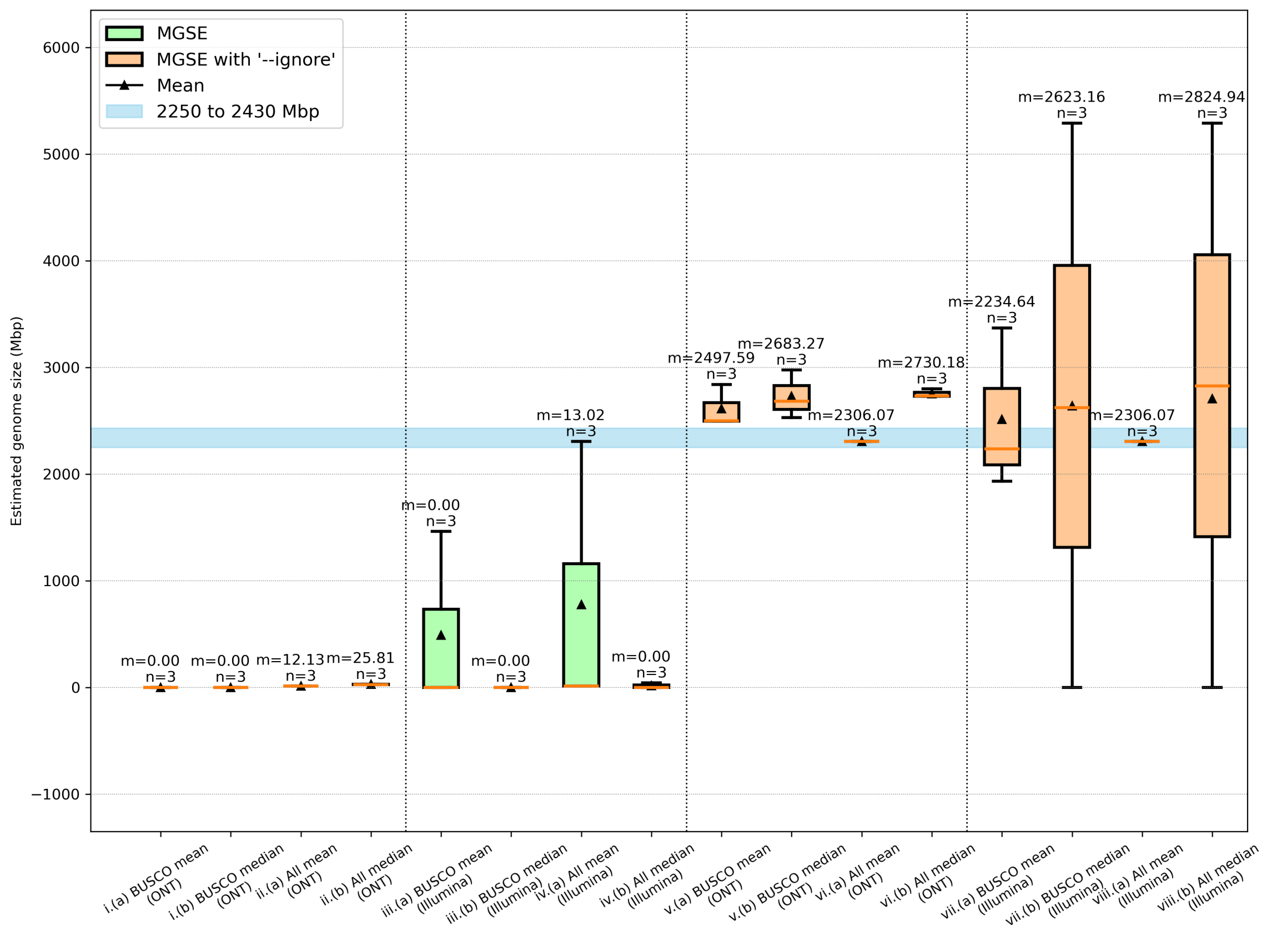

Supplement: Supplementary file 14 — Supplementary Material 14 [file 12864_2025_11640_MOESM14_ESM.pdf]
